# Secondary prevention of peanut allergy – continuous feeding or restriction of peanut protein

**DOI:** 10.1186/2045-7022-3-S3-P139

**Published:** 2013-07-25

**Authors:** J Bellach, B Niggemann, K Beyer, B Ahrens

**Affiliations:** 1 Department of Pediatrics, Division of Pneumology and Immunology, Charité Universitätsmedizin, Berlin, Germany; 2 Department of Pediatrics, DRK-Kliniken Westend, Berlin, Germany

## Background

Peanut Allergy (PA) is one of the major causes for severe allergic reactions. Most children with PA also suffer from atopic eczema (AE). So far strategies for PA prevention are not established. The aim of our ongoing study is to compare if continuous feeding or a strict avoidance of peanut protein may prevent children with AE for the development of PA.

## Methods

In an open study design 460 children aged 5 to 30 months with AE and no detectable peanut-specific serum IgE will be divided into two groups. In *Group A* parents are advised to feed their children peanut at least three times a week, in *Group B* participating children strictly avoid peanut in their diet. Children are followed for one year. At the beginning and the end of the study standardized questionnaires are performed, blood samples are collected and medical examination (including SCORAD) is assessed. Children who will develop sensitization to peanut will undergo a double blind placebo controlled food challenge.

## Results

So far 142 children are included. 68 parents decided for *Group A* and 74 parents for *Group B*. The average age of the children in *Group A* is 19 months (ranging from 5 to 29 months) and in *Group B* 22 months (ranging from 6 to 28 months). The SCORAD assessed at the time of the first visit accounted 31 (ranging from 10 to 76) and 26 (ranging from 13 to 81) in *Group A* and *Group B*, respectively.

## Conclusion

Many parents reported uncertainty about the current dietary recommendations. Despite the fact that the study is planned as an open study and parents can decide whether to feed peanut or not the decision for either group appears to be equal so far. The finalization of our study will hopefully contribute to improved PA prevention strategies.

## Disclosure of interest

None declared.

